# Pharmacokinetics in children with chronic kidney disease

**DOI:** 10.1007/s00467-019-04304-9

**Published:** 2019-08-02

**Authors:** Anne M. Schijvens, Saskia N. de Wildt, Michiel F. Schreuder

**Affiliations:** 1 Radboud Institute for Molecular Life Sciences, Department of Pediatric Nephrology, Radboud University Medical Center, Amalia Children’s Hospital, P.O. Box 9101, 6500 HB Nijmegen, The Netherlands; 2 Department of Pharmacology and Toxicology, Radboud University Medical Center, Nijmegen, The Netherlands; 3 Intensive Care and Department of Pediatric Surgery, Erasmus MC Sophia Children’s Hospital, Rotterdam, The Netherlands

**Keywords:** Pharmacokinetics, CKD, Absorption, Distribution, Metabolism, Excretion, Children

## Abstract

In children, the main causes of chronic kidney disease (CKD) are congenital diseases and glomerular disorders. CKD is associated with multiple physiological changes and may therefore influence various pharmacokinetic (PK) parameters. A well-known consequence of CKD on pharmacokinetics is a reduction in renal clearance due to a decrease in the glomerular filtration rate. The impact of renal impairment on pharmacokinetics is, however, not limited to a decreased elimination of drugs excreted by the kidney. In fact, renal dysfunction may lead to modifications in absorption, distribution, transport, and metabolism as well. Currently, insufficient evidence is available to guide dosing decisions on many commonly used drugs. Moreover, the impact of maturation on drug disposition and action should be taken into account when selecting and dosing drugs in the pediatric population. Clinicians should take PK changes into consideration when selecting and dosing drugs in pediatric CKD patients in order to avoid toxicity and increase efficiency of drugs in this population. The aim of this review is to summarize known PK changes in relation to CKD and to extrapolate available knowledge to the pediatric CKD population to provide guidance for clinical practice.

## Introduction

Chronic kidney disease (CKD) is a general term for multiple, heterogeneous disorders causing irreversible kidney damage, which is a major public health problem worldwide [1]. The overall prevalence in children ranges from 55 to 75 per million [2, 3]. The causes of CKD in children are very different from adults. In fact, in adults, diabetic nephropathy and hypertension are the main causes of CKD, whereas CKD in children is often caused by congenital diseases and glomerular disorders [2, 3].

**Fig. 1:**
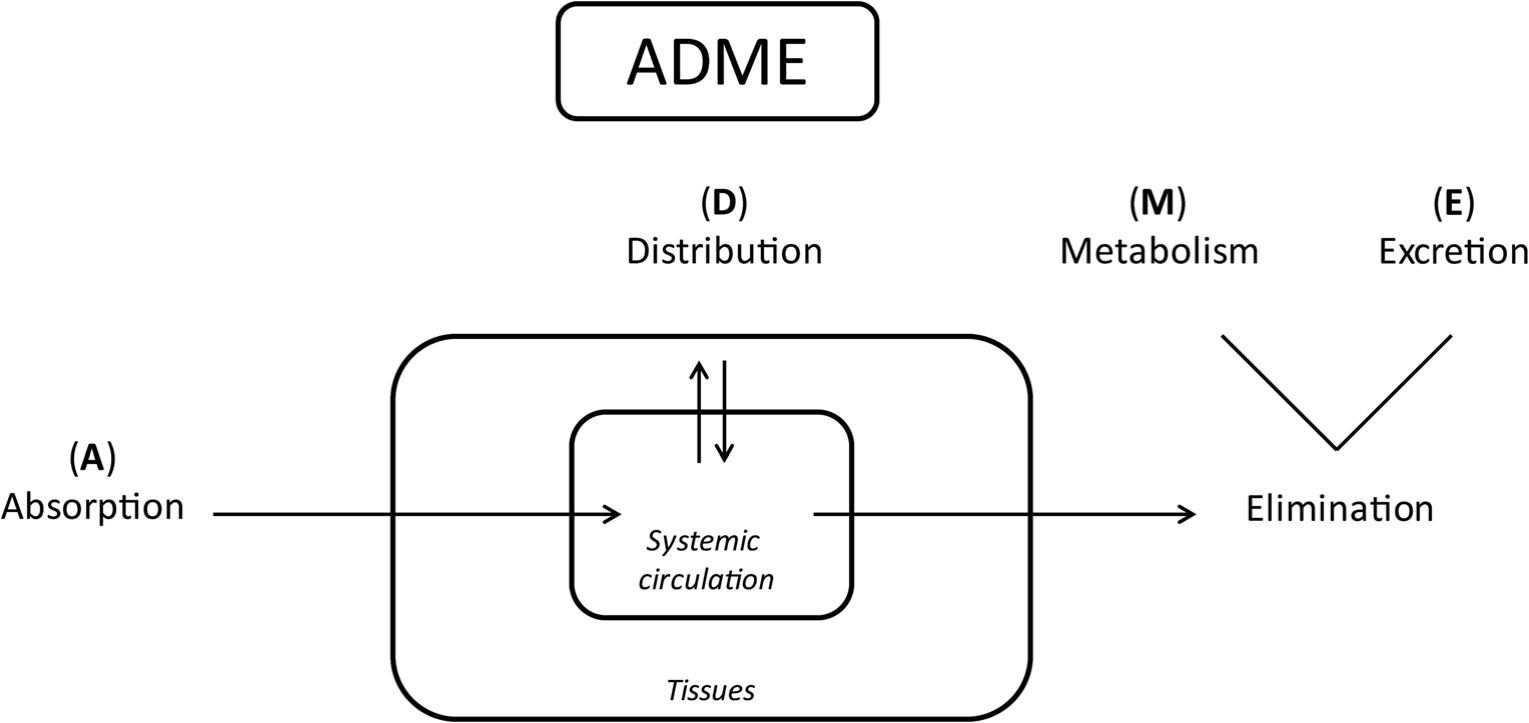
Overview of pharmacokinetic processes. ADME absorption, distribution, metabolism, and excretion

The kidneys play an important role in handling of drugs, most importantly in excretion. A well-known consequence of CKD on pharmacokinetics (PK) is a reduction in renal clearance due to a decrease in the glomerular filtration rate (GFR). The impact of renal impairment on the PK of drugs is, however, not limited to a decreased elimination of drugs excreted by the kidneys. PK describes the individual steps that determine drug disposition in the body, namely absorption from an extravascular site of administration, distribution to various tissues, and elimination from the body based on metabolism and excretion (Fig. 1). In fact, CKD is associated with multiple physiological changes and may therefore influence extrarenal PK processes, which may increase the risk of toxicity [4–6]. Consequently, patients with impaired kidney function are more at risk of altered drug exposure or toxic effects than individuals with normal kidney function [7].

Drug dosage adjustment guidelines, based on the assumption that systemic clearance primarily reflects renal clearance and is proportional to kidney function, are commonly used. However, response to drug therapy is often less predictable, which is illustrated by the fact that the frequency of adverse drug reactions and other medication-related problems is higher in patients with kidney disease than in those with normal kidney function [8, 9]. Despite numerous published guidelines regarding drug dosing for patients with reduced kidney function, there is insufficient evidence to guide decisions on many commonly used drugs [10]. Moreover, the impact of maturation on drug disposition and action should be taken into account when dosing drugs in children. Evidence on the impact of growth and development on absorption, distribution, metabolism, and excretion (ADME) of drugs has increased significantly over the years [11]. However, the exact interplay between age and disease on PK, pharmacodynamics (PD), and dose requirements remains poorly understood [11, 12]. Furthermore, the majority of drugs prescribed in children are off-label [13, 14], which limits the evidence on drug dosing in pediatric CKD patients even further. Clinicians should take PK changes into consideration when selecting and dosing drugs in children with CKD in order to avoid toxicity and to increase efficiency of drugs in this population. Unfortunately, very little information is available on PK changes in pediatric CKD patients. Therefore, data from animal studies, non-CKD children, and clinical studies in adult CKD patients are used to get an appreciation of the possible impact of these conditions on the PK in pediatric patients. The aim of this review is to summarize known PK changes in relation to CKD with respect to ADME and extrapolate available data to the pediatric CKD population to provide knowledge for clinicians prescribing drugs in this vulnerable population.

## Absorption

Absorption describes the extent to which an intact drug is absorbed after oral administration from the gut lumen into the portal circulation. Several factors are known to have an impact on absorption, such as dissolution of the drug, the gastric emptying rate, gastric pH, intestinal motility, drug interactions, and passage through the gut wall [15]. Some of these factors may vary with growth and development. Ultimately, this may result in changes in the drug absorptive capacity at different ages in the individual pediatric patient [11]. Maturational changes in the gastrointestinal tract were reviewed by Neal-Kluever et al. [16] and Mooij et al. [17]. The absorption and bioavailability of drugs are highly variable in patients with CKD, in whom several pathophysiological changes in the gastrointestinal tract have been identified that may impact drug absorption [4]. Thus far, only little research has been conducted to investigate the influence of CKD on drug absorption in children.

### Gastric emptying

#### Impact of age in non CKD children

Bonner et al. investigated the impact of age and other covariates on the rate of gastric emptying by analyzing published data on approximately 1500 individuals ranging from premature neonates to adults. A model-based meta-analysis indicated that age itself is not a covariate of gastric emptying [18]. Similarly, Billeaud et al. showed that in children between 0 and 1 year old, gastric emptying did not vary with age [19]. On the contrary, Anderson et al. reported slow absorption of acetaminophen in neonates, with a significant increase in the first days of life, suggesting fast maturation of gastric emptying in early life [20]. Furthermore, gastric emptying appears to be slower in preterm neonates compared to term neonates [21].

#### Adult CKD patients

Patients with CKD may suffer from delayed gastric emptying for which several patient characteristics and external factors can be identified, including peritonitis, peritoneal dialysis, and pharmacotherapy (e.g., aluminum containing antacids, opioids) [6, 22]. Results on gastric emptying in adult CKD patients are conflicting, ranging from no obvious impairment to a significant delay in over 30% of the population [23–25]. Ultimately, decreased gastric emptying in CKD patients affects the time to reach the maximum drug concentrations (Tmax) and peak plasma drug concentration (Cmax) but is not expected to have an impact on bioavailability [26, 27].

#### Pediatric CKD patients

Ravelli et al. investigated gastrointestinal function in 12 pediatric CKD patients and found both delayed (*n* = 5) as well as accelerated (*n* = 2) gastric emptying [28]. Furthermore, Ruley et al. showed that gastroesophageal reflux, as a manifestation of gastrointestinal dysmotility, was present in 73% of the pediatric CKD patients [29].

### Gastric pH

#### Impact of age in non CKD children

Changes in the gastric pH may influence the bioavailability of many drugs. In children, gastric pH is neutral at birth due to fetal ingestion of alkaline amniotic fluid [30]. In the first few hours after birth, after amnion fluids are removed from the stomach, a rapid decrease in pH is noticed, most likely explained by gastric secretion [30]. Generally, as reviewed by Mooij et al., mean gastric pH remains around 2 or 3 in children of all ages [17]. Gastric pH rises after feeding; however, as pH rapidly decreases again and most children receive intermittent feeding, the effect on absorption of acid-labile drugs is limited [31]. On the contrary, one may hypothesize that in children with very frequent or continuous milk-based feeding regimens, acid-labile drugs may be absorbed more efficiently due to a persistent higher gastric pH.

#### Adult CKD patients

CKD patients often have an increased gastric pH, which may have several causes. For instance, patients with renal dysfunction have increased blood urea nitrogen. Excess salivary urea is converted to ammonia by gastric urease enzymes, resulting in increased gastric pH [32, 33]. Furthermore, patients are often treated with antacids, H2-receptor antagonists, or proton-pump inhibitors that alter gastric pH [34]. The resulting increase in gastric pH may affect the ionization and dissolution of drugs that are soluble in acidic environments, like furosemide or iron therapy, and reduce their bioavailability by approximately 20% and 50%, respectively [35, 36].

#### Pediatric CKD patients

Currently, studies on gastric pH in children with CKD are limited. However, feeding problems, anorexia, and recurrent vomiting are prevalent problems in pediatric CKD patients [37, 38]. Ravelli et al. investigated the percentage of time spent with an intraesophageal pH below 4 and found significantly higher mean values in pediatric CKD patients compared to age-matched controls. Concomitant drug use was, unfortunately, not reported in these patients [28].

### Formation of insoluble salts or metal ion chelates

Some of the drugs administered in patients with CKD may alter the absorption of other drugs. The ingestion of cation-containing antacids (e.g., sevelamer hydrochloride, lanthanum carbonate) and minerals (e.g., calcium, magnesium) may reduce drug absorption because of chelation with co-administered medications, resulting in the formation of insoluble salts or metal ion chelates [26, 39]. For example, bioavailability of oral ciprofloxacin was significantly decreased when co-administered with sevelamer hydrochloride or calcium acetate by 48% and 51%, respectively, due to formation of chelate complexes [39, 40].

### Intestinal transport and metabolism

After ingestion, the drug reaches the lumen of the gut, where it may either diffuse passively or be actively transported by uptake transporters across the apical membrane into the enterocyte. Once inside the enterocyte, drugs can be actively excreted by transporters or metabolized by intestinal enzymes [41]. The most important drug metabolizing enzyme family is the cytochrome P450 (CYP) family, with the CYP3A4 isoenzyme as the most prevalent drug-metabolizing enzyme. The CYP3A subfamily is present in the intestine, more specifically in the villi, in abundance and contributes to the first-pass metabolism of several CYP3A4 substrates such as midazolam, cyclosporine, and tacrolimus [42–45]. In addition to the intestine, other organs, including the liver, contain a diversity of drug metabolizing enzymes (DMEs) as well [43, 44]. More detailed information regarding DMEs is given in the metabolism section of this review.

Drug transporters are transmembrane proteins facilitating the passage of both drugs and other xenobiotics across biological barriers [46]. Transporters are characterized as either influx transporters, which facilitate transport into the cell, or efflux transporters, facilitating the transport out of the cell. The presence of transporters is not limited to the gut [47]. In fact, multiple uptake and efflux transporters are expressed in the membranes of the intestines, liver and kidneys (for reviews on these transporters, see [48, 49]). An example of an important drug efflux transporter in the gastrointestinal tract and hepatobiliary system is P-glycoprotein (P-gp). P-gp is an adenosine triphosphate (ATP)-dependent efflux pump expressed on the apical membrane of tissues, which are often exposed to high concentrations of xenobiotics. The function of P-gp is to protect the body against toxic compounds by transporting those out of the cell and the body via the intestinal lumen, bile, or urine [50, 51]. Conversely, organic anion-transporting polypeptides (OATP) are a group of uptake transporters expressed on the basolateral surface of membranes with a similar tissue distribution to P-gp [46]. OATP mediate the transport of mainly organic anions across the cell membrane into the cell.

#### Impact of age in non CKD children

In general, little is known about transporter gene expression in children. Furthermore, evidence on the ontogeny of the different influx and efflux transporters in children is limited [11, 52]. The ontogeny of expression of P-gp was investigated in 59 normal duodenal biopsies of children aged 1 month to 17 years. Fakhoury et al. found P-gp mRNA expression levels to be highly variable and unrelated to age [53]. In addition, Mooij et al. observed stable intestinal P-pg mRNA expression from neonatal to adult age, confirming the aforementioned finding [54]. In contrast, multidrug resistance-associated protein 2 (MRP2), another intestinal efflux transporter, showed a different pattern, with similar mRNA expression levels in neonates and adults but significantly decreased levels in children aged 1–12 months [54]. Furthermore, intestinal OATP2B1 mRNA expression levels were higher in neonates compared to adults. In children with an age range of 1–12 months, mRNA expression levels reached adult values [54]. To our knowledge, no in vivo studies have been conducted to investigate activity of intestinal drug transporters in pediatric patients. Drug metabolizing activity may change significantly from fetal to adolescent age. Data regarding the expression of CYP3A enzymes in the gut wall in children are contradictory, ranging from an increase with age to the opposite pattern of a decrease with age. Johnson et al. investigated enterocytic CYP3A expression in duodenal biopsies in fetuses and children (age range 2 weeks–17 years) and found a significant increase in CYP3A4 expression and activity with age [55]. In contrast, another study showed high CYP3A4 mRNA expression levels in the first year of life, followed by a decrease with age [53]. Using a physiological population PK modeling approach, Brussee et al. simulated intestinal CYP3A4 activity per gram of organ to remain relatively constant throughout childhood, indicating that organ growth appears the most important contributing factor to the increase in intrinsic CYP3A clearance in the gut wall [56]. Using a similar approach, low CYP3A activity in the gut wall was found in preterm neonates, yielding a low first-pass effect and higher bioavailability in this patient group compared to adults [57]. The ontogeny of other intestinal drug-metabolizing enzymes is still largely unknown.

#### Animal studies

CKD may increase bioavailability of drug substrates due to downregulation of transporters and enzymes in the enterocytes. A decrease in intestinal drug efflux activity may lead to increased bioavailability and increased systemic exposure of various drugs, such as calcineurin inhibitors [5]. Evidence supporting this phenomenon is mostly based on animal studies using CKD models [58, 59]. Leblond et al. showed that CKD in rats is associated with a decrease in intestinal CYP1A1 and CYP3A2 activities [60], whereas intestinal CYP3A function was investigated in several clinical studies by using phenotyping probes and appeared not to be substantially altered in patients with end-stage kidney disease (ESKD) [61–63]. Veau et al. found a reduction in intestinal drug elimination in CKD rats due to a significant decrease in P-gp transport activity without a decrease in protein expression [59]. Moreover, Naud et al. showed a significant reduction in both transport activity as well as protein expression of intestinal P-gp in CKD rats [58].

#### Pediatric CKD patients

**Fig. 2:**
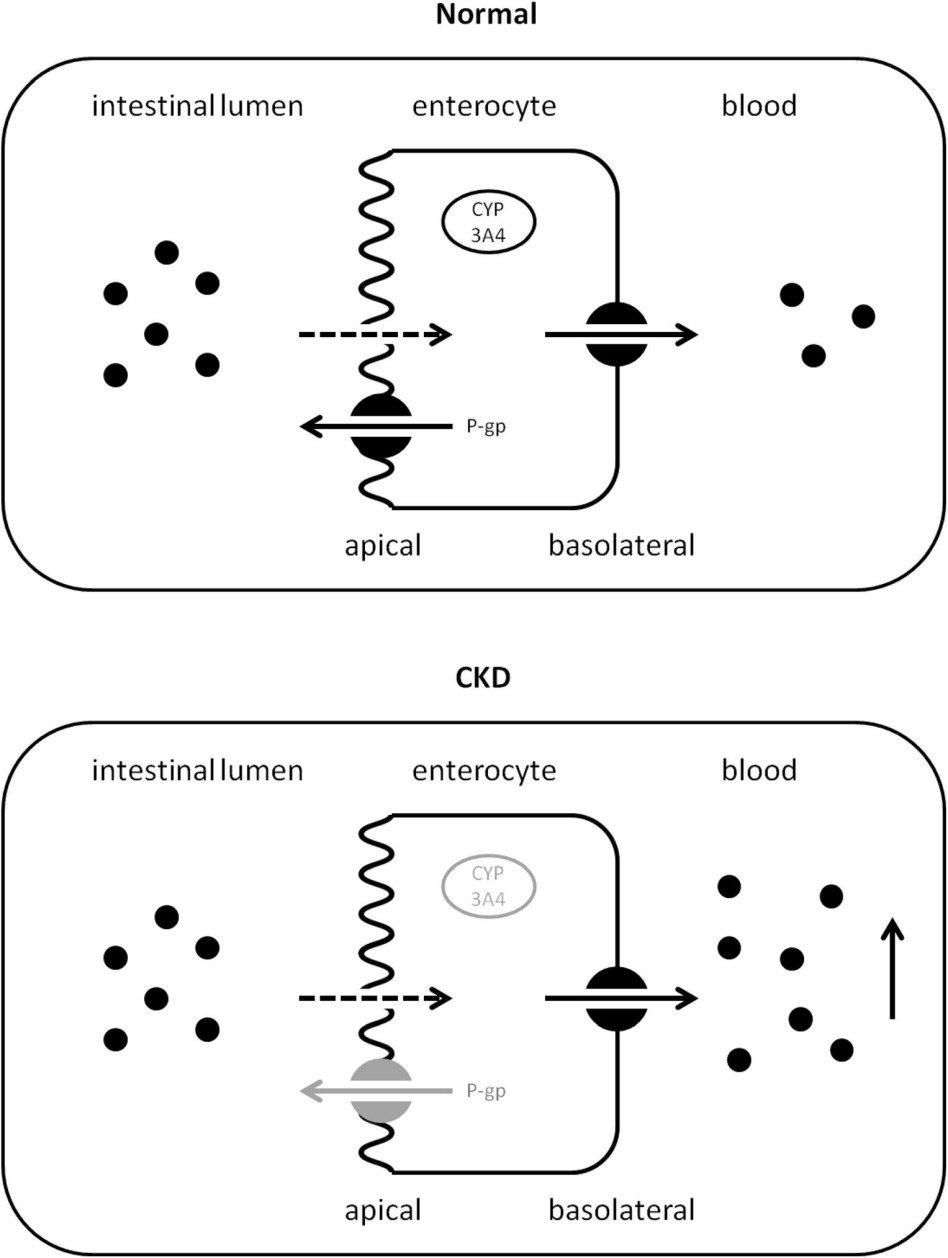
Impact of CKD and diarrhea on tacrolimus bioavailability. CKD chronic kidney disease, CYP cytochrome P450 enzyme

A frequently observed interaction in pediatric nephrology is the effect of diarrhea on tacrolimus levels in kidney transplant recipients. Tacrolimus is extensively metabolized by CYP3A4 and is a substrate for P-gp. Oral bioavailability of the drug is low, due to metabolism in the small intestine by CYP3A4 and active secretion into the gut lumen by P-gp [64]. The concentration of CYP3A4 enzymes decreases from the duodenum to the colon. In case of severe diarrhea, the gastrointestinal transit time is decreased. This could be an explanation for an increased oral tacrolimus bioavailability as the drug is shunted to the colon with lower intestinal metabolism [65]. Furthermore, the epithelial cells of the intestine may be damaged during the course of diarrhea. This may reduce the enzymatic activity of CYP3A4 and/or P-gp in the enterocytes and will subsequently lead to increased levels of tacrolimus [66, 67] (Fig. 2). Taken together, CKD-induced reduction in intestinal metabolism and P-gp-mediated drug transport might result in increased oral bioavailability of certain drugs. Therefore, a decrease in dose may be necessary for drugs that are substrates for P-gp and/or CYP3A4 [5, 68].

### Bowel wall edema

GI edema has also been identified as a potential cause of altered drug absorption, particularly in CKD patients with concomitant cirrhosis or congestive heart failure [6, 69]. Bowel wall edema increases intestinal permeability and may therefore impair the intestinal barrier function in CKD patients [69].

## Distribution

After absorption, drugs distribute to target tissues and sites of elimination in the systemic circulation. The volume of distribution (Vd) represents the parameter relating the concentration of a drug in the plasma to the total amount of the drug in the body. Several physiologic variables may affect the Vd, including physicochemical properties of the drug (e.g., size, charge, acid dissociation constant, water solubility, lipid solubility), plasma protein binding, tissue binding, and total body water. With the exception of the physicochemical properties of the drug, these variables may be affected in children with CKD [4]. Unfortunately, no clinical data are available in pediatric kidney transplant patients. Therefore, age-related changes in non CKD children and clinical data in adult CKD patients are summarized below.

### Protein binding

Many drugs are extensively bound to plasma proteins, and the Vd is highly dependent on the protein binding of the drug. Protein binding limits drug distribution as only the unbound concentration of the drug is able to cross cellular membranes and distribute outside the vascular space and is therefore pharmacologically active [41]. The major drug binding proteins in plasma are albumin and alpha1-acid glycoprotein (AAG). Acidic drugs are bound to albumin, whereas alkaline drugs primarily bind to AAG. AAG is an acute phase protein with one binding site for alkaline drugs.

#### Impact of age in non CKD children

Children generally have lower concentrations of the important binding proteins, which is most pronounced in newborns and young infants [70]. Furthermore, in newborns, fetal albumin (with a reduced binding affinity for weak acids) and endogenous substances, such as bilirubin, are present. This contributes to higher free fractions of highly protein-bound drugs, due to the capacity of these substances to displace a drug from the albumin binding sites [71]. Clinical implications are especially present for highly protein-bound drugs with a narrow therapeutic index, such as vancomycin [72].

#### Adult CKD patients

**Fig. 3:**
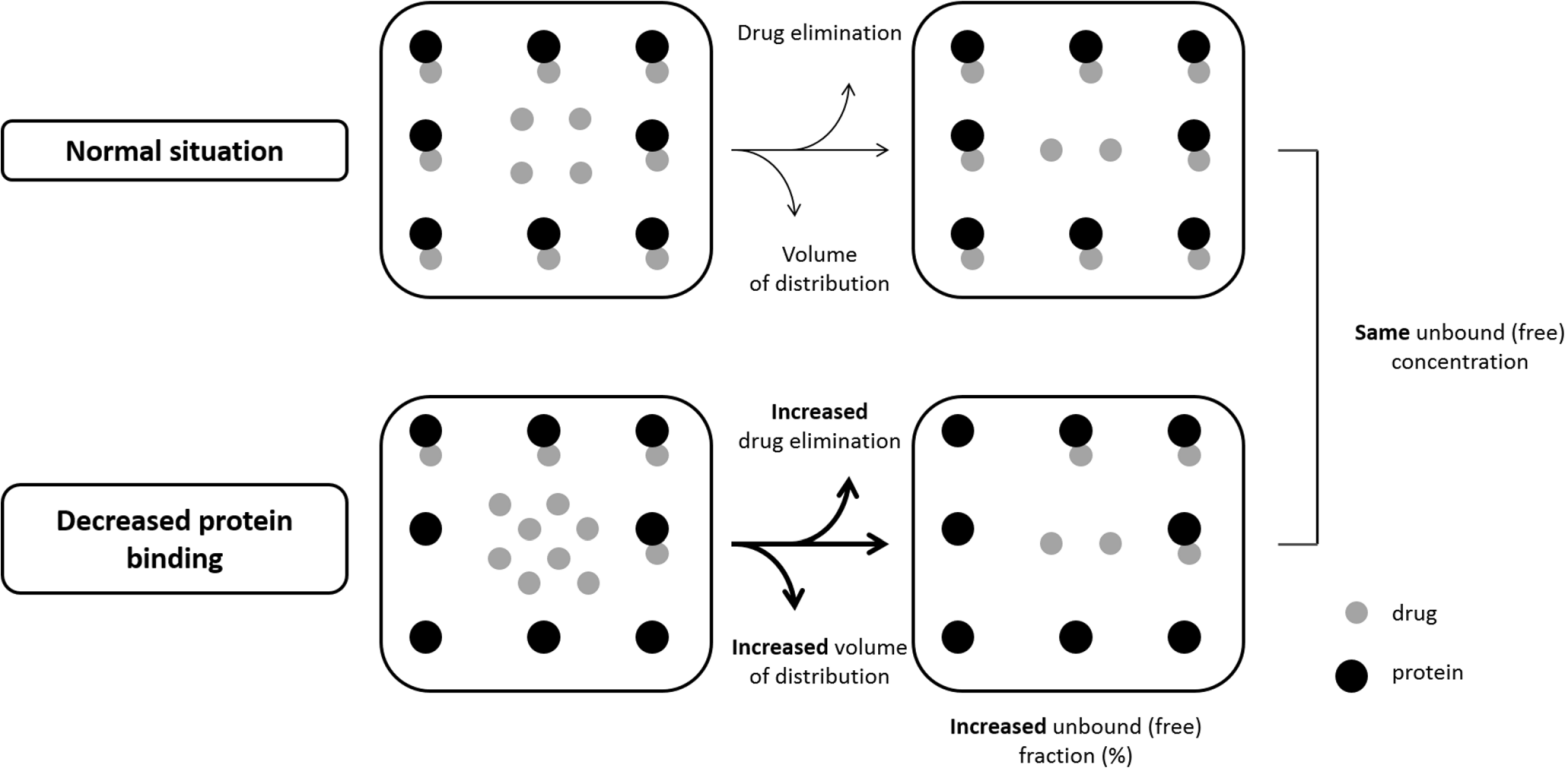
Decreased protein binding in CKD patients

AAG is reported to be increased up to three times in CKD patients and patients on dialysis as a result of chronic inflammation [34, 73, 74]. In line with this, an increased plasma binding of alkaline drugs, such as propranolol and cimetidine, has been demonstrated in vitro [75]. In vivo AAG binding, however, generally appears to be unaffected in patients with CKD [34, 75–77]. Plasma protein binding of acidic drugs, such as penicillins, cephalosporins, furosemide, and phenytoin, is often decreased in vivo in CKD patients [34, 77]. This decrease has been suggested to be due to proteinuria- or malnutrition-related low plasma albumin, conformational change of the albumin binding sites due to uremia, or the accumulation of competitive inflammatory factors, protein-bound uremic toxins, and/or drug metabolites competing with the acidic drugs for protein binding sites [78, 79]. The last factor appears, however, to be most important [80]. A decrease in protein binding leads to an increase in the unbound fraction of the drug. Generally, this has no significant clinical implications as the unbound drug is readily available for elimination and distribution in tissues, leading to increased clearance and Vd (Fig. 3). The overall effect is a new steady-state situation in which the concentration of unbound drug and therefore pharmacological effect is unaffected [81]. This phenomenon can be illustrated by the distribution of phenytoin in CKD patients. Phenytoin is highly protein-bound (90%) in healthy and around 80% in CKD patients [82], but the pharmacologically active, unbound concentration in plasma is unaffected. The decreased total plasma concentration could be misinterpreted as a need for dose correction; subsequently, the increase in dose may produce toxicity with no increased effectiveness [75]. Ideally, free concentrations should therefore be monitored for highly protein-bound drugs with narrow therapeutic indices in CKD patients.

### Tissue binding

Vd may also be affected by altered tissue binding, e.g., in patients with ESKD. The Vd of digoxin can be reduced by 50% due to decreased tissue binding [83–85], potentially due to a reduction in tissue levels of Na/K-ATPase, the major tissue-binding site for digoxin [86]. The reduction in Vd may result in increased serum concentrations if the loading dose is not reduced. However, the pharmacological effect of digoxin correlates with the amount of drug in the myocardium and Jusko et al. reported the myocardium-to-serum concentration ratio of digoxin to decrease in parallel with renal clearance [84]. Therefore, a decrease in loading dose may not be necessary. Moreover, as toxic effects also rely on the presence of tissue binding sites, increased serum concentrations may not lead to toxic effects [87].

### Fluid retention

CKD may cause severe changes in body composition. Furthermore, body composition changes with age and may affect the physiological spaces into which a drug will distribute [12]. An already physiological large total body water compartment in a neonate, combined with a higher total body water due to CKD, could result in a significantly increased Vd [13]. Excessive fluid retention, manifesting as increased extracellular fluid such as edema or ascites, is expected to increase the Vd of hydrophilic drugs. An increase in extracellular fluid volume will have the greatest effect on hydrophilic drugs with low to moderate Vd (i.e., < 0.7 L/kg), such as aminoglycosides and cephalosporins, resulting in lower plasma and tissue concentrations [68]. However, as aminoglycosides and cephalosporins are largely excreted unchanged in the urine, a decrease in kidney function also causes a prolonged half-life and will lead to increased drug concentrations.

## Metabolism

Nonrenal clearance includes all routes of drug elimination, including metabolism, except for renal excretion of unchanged drugs. In fact, only a few drugs are excreted unchanged by the kidney. Metabolism is the major mechanism for elimination of drugs from the body [88]. Drug metabolism is classified as either a phase I or a phase II reaction. The major enzymes responsible for phase I metabolism are the CYP enzymes [89]. The most abundant CYP enzyme, CYP3A, is responsible for the metabolism of many drugs [90]. The most important groups of DMEs in phase II metabolism are the superfamily of uridine 5′-diphospho-glucuronosyltransferases (UDP-glucuronosyltransferases, UGT). For comprehensive information regarding CYP substrates, inhibitors, and inducers, see Flockhart table [91].

### Hepatic metabolism

CKD may have various effects on the metabolism of drugs. Decreased protein expression, mRNA expression, and/or activity of several nonrenal clearance pathways have been reported in experimental animal models of CKD [92, 93]. Decreased functional expression of hepatic DMEs could lead to a reduction in hepatic clearance of relevant substrates. Thus far, the exact mechanism by which CKD may affect PK of nonrenally eliminated drugs is not entirely understood. However, the most important hypothesis is direct inhibition of non renal clearance pathways by accumulated uremic toxins. Due to kidney failure, molecular breakdown products, which are normally eliminated by the kidneys, now accumulate in the body. These molecular breakdown products include urea, inflammatory cytokines, and indoxyl sulfate, also known as uremic toxins [94, 95]. Uremic toxins cause downregulation of gene expression mediated by proinflammatory cytokines and directly inhibit the activity of CYP enzymes and drug transporters.

#### Impact of age in non CKD children

Maturational changes are well known to occur in the DMEs and have a clear impact on drug disposition in children [96]. Age-dependent changes are enzyme and organ specific. For instance, hepatic CYP3A7 is present at birth and almost disappears after infancy [97]. In contrast, CYP3A4 appears in the first week of life and reaches 30–40% of adult activity after 1 month [97]. CYP3A4 reaches an adult level of activity at the end of childhood, while CYP3A5 activity appears stable, but with large genetic variation [90, 96]. While the ontogeny of hepatic phase I metabolism is increasingly known, our knowledge on phase II metabolism lags behind [98]. A well-known example of UGT maturational change is the development of the potentially lethal gray baby syndrome in neonates receiving chloramphenicol, which is a consequence of accumulation in the body due to immature glucuronidation by UGT2B7 [99, 100]. Similarly, neonatal glucuronidation of morphine (a UGT2B7 substrate) is decreased in newborns compared with adolescents [101, 102]. For additional reviews on metabolism including ontogeny of DMEs and age-related changes in metabolism of drugs, please see [103, 104].

#### Animal studies

The expression and activity of CYP3A in CKD patients have been studied in several experimental models and clinical studies [93, 105]. Leblond et al. showed a significant decrease in total liver CYP activity (mainly in CYP2C11, CYP3A1, and CYP3A2) secondary to reduced gene expression in rats with CKD [106, 107]. Furthermore, phase II DMEs may also be affected by CKD. For example, Simard et al. showed a decrease in *N*-acetyltransferase (Nat)1 and Nat2 proteins and Nat2 activity secondary to a decrease in gene expression in CKD rats causing a decrease in drug acetylation [108].

#### Adult CKD patients

Yoshida et al. investigated the effect of CKD on the PK of in vivo model drugs of CYP3A4/5 in humans and found a modest but variable effect [92]. The effect of CKD on the expression of CYP3A, however, might be a reflection of changes in transporter function rather than a change in enzyme activity itself. This hypothesis is supported by the fact that the PK of midazolam, a CYP3A substrate neither a P-gp nor an OATP substrate, is not altered in CKD patients [63]. In contrast, in vivo CYP2B6, CYP2C19, CYP2D6, and CYP2E1 decreased in parallel with the degree of CKD [92, 109]. Furthermore, changes to CYP1A2, CYP2C9, and CYP2C8 due to CKD appear limited [110, 111]. Osborne et al. showed a significant increase in the area under the curve of morphine in CKD patients compared to control subjects, which suggests reduced UGT2B7 activity, but a role of the OCT1 transporter cannot be excluded [112].

## Excretion

### Biliary excretion

Biliary excretion eliminates substances from the body when the secreted drug is not reabsorbed from the intestine (enterohepatic cycle). Little is known about the developmental changes in biliary excretion in children [113]. Alterations in various biliary efflux transporters have been found in experimental models of CKD. An increase in the expression of hepatic efflux transporters, including P-gp, was reported resulting in an increase in biliary excretion [114]. In contrast, the protein expression of uptake transporter OATP2 was found to be decreased in animal studies, causing a reduction in biliary and metabolic clearance [114]. This was confirmed in vivo by Nolin et al. [63].

### Renal excretion

Only few drugs are excreted almost entirely unchanged by the kidney, for instance, aminoglycosides and penicillins [115, 116]. Renal drug clearance is the net result of three processes: filtration at the glomerulus, active secretion and reabsorption by the proximal tubule, and passive reabsorption in the kidney tubules. According to the “Intact Nephron Hypothesis,” all segments of the nephron are equally affected by the development of any type of renal disease [77, 117]. This suggests that, regardless of the intrarenal pathways of excretion, the loss of excretory function in the diseased kidney can be quantified by GFR. However, depending on the cause of renal dysfunction, the normal histology of the glomeruli and the tubules may be differentially affected [118].

### Glomerular filtration

As blood passes through the glomerulus (± 1000 ml/min in an average adult), about 20% of the plasma is filtered into the renal tubule (GFR 120 ml/min). Furthermore, the unbound drug in plasma water is filtered as well, whereas drugs bound to plasma proteins are not filtered. Glomerular filtration depends on kidney blood flow, which can decrease when a reduced cardiac output or volume depletion is present [5]. GFR is often used as an indicator of overall kidney function.

#### Impact of age in non CKD children

At the transition from fetal to extrauterine life, the glomerular filtration needs to develop. By 36 weeks of gestation, nephrogenesis is complete [119]. After birth, nephrons are slowly recruited as reviewed by Filler et al. [120]. In term neonates, GFR is just 2–4 ml/min/1.73 m^2^; it doubles by 1–2 week(s) of age, reaching adult values at approximately 12–24 months of age [121, 122]. Furthermore, GFR continues to increase after reaching adult values until prepubescent age, resulting in a higher clearance compared to adults [123, 124]. The development of GFR is slowed in preterm born neonates, even though normal values are reached in the end [121]. Maturation of the glomerular filtration and the different patterns depending on perinatal circumstances on this maturation has a major impact on renal drug clearance.

#### Adult CKD patients

Endogenous creatinine clearance is frequently used as a measure for GFR. However, a discrepancy may be present between endogenous creatinine clearance and GFR, which is most pronounced in subjects with low GFR. This is due to an increasing tubular secretion of creatinine with increasing serum creatinine [125, 126]. In that case, creatinine clearance overestimates GFR. Moreover, muscle mass is typically decreased in patients with severe renal dysfunction, leading to a reduced production rate [126]. Furthermore, tubular secretion of creatinine can be inhibited by various drugs. Examples of drugs that inhibit creatinine secretion include the following: triamterene, spironolactone, amiloride, and trimethoprim [127–129]. In general, the GFR is decreased in CKD patients. According to the Intact Nephron Hypothesis, all segments of the nephron are equally affected by the development of any type of renal disease [77, 117]. This suggests that, regardless of the intrarenal pathways of excretion, the loss of excretory function in the diseased kidney can be quantified by GFR. However, depending on the cause of renal dysfunction, the normal histology of the glomeruli and the tubules may be differentially affected [118].

In CKD patients, not only the drug itself may accumulate; accumulation of drug metabolites that are primarily excreted by the kidneys may also be an issue [130, 131]. Due to a decrease in GFR, patients will be exposed to prolonged drug effects or even toxicity if the metabolites are pharmacologically active, especially if a large percentage of the active metabolite is excreted unchanged by the kidney under normal circumstances. A well-known example of this phenomenon is the administration of morphine in CKD patients. Renal excretion of morphine itself only accounts for approximately 4% of its overall elimination. However, patients with renal dysfunction may show typical signs of morphine intoxication when given standard doses of morphine. Studies have shown that the major morphine metabolites, which are normally excreted by the kidney, extensively accumulate in patients with renal dysfunction [132, 133]. Thus, despite the fact that the kidneys are only marginally involved in the elimination of morphine, patients with CKD can still show signs of morphine intoxication due to accumulation of active metabolites. Moreover, uremic toxins can compete with acidic drugs for active secretion by the kidney [134].

### Active tubular secretion

In the proximal tubule, several transporters are present to facilitate both tubular secretion and tubular reabsorption of drugs, exogenous, and endogenous substances. Transporters are localized at the basolateral and apical membranes of the proximal tubular epithelial cells [135]. For some compounds, active secretion is significant and therefore the renal clearance exceeds the GFR. This is the case with creatinine, but this can also occur with drugs, such as metformin and amoxicillin [136, 137].

#### Impact of age in non-CKD children

As reviewed by Brouwer et al., little evidence is available on the ontogeny of drug transporter expression in the developing human kidney. However, from both animal and human studies, one may conclude that renal transporters appear to mature at different rates [113]. Para-aminohippurate clearance, a substrate of the organic anion transporter 1 (OAT1), was low at birth, with an increase in the first weeks of neonatal life, reaching adult levels around 1 year of age [138–140]. Similarly, Momper et al. reanalyzed previously published data on the maximum tubular secretory capacity of PAH (TmPAH) from 119 neonates, infants, and children. TmPAH was low in the immediate postnatal period, increased markedly after birth, and reached 50% of the adult value at 8 years of age [141]. While PAH clearance may reflect OAT1 maturation, it cannot be excluded that these findings can also be explained by maturation in renal blood flow, as PAH is also a marker of renal blood flow. Digoxin is excreted by glomerular filtration and extensively secreted in the proximal tubule by P-gp. Pinto et al. investigated age-dependent expression of renal P-gp in mice and its correlation with changes in the clearance rate of digoxin. A significant correlation between P-gp expression and digoxin clearance values was found [142]. In line with these results, young children need significantly higher doses of digoxin per kilogram of body weight than adults. This cannot be explained by GFR changes alone and may indicate higher renal P-gp expression in young children than in adults.

#### Animal studies

Komazawa et al. investigated transport activity of renal transporters in CKD rats and showed a decrease in tubular function in line with a decrease in glomerular filtration. Expression levels of organic anion transporter (Oat)1, Oat3, organic cation transporter (Oct)1, and Oct2 were found to be decreased in CKD rats. In contrast, levels of P-gp were significantly increased [143]. Similarly, Naud et al. also examined the effects of CKD on the expression and activity of the major renal drug transporters in rats. A significant correlation was found between the clearance of creatinine and the protein expression of transporters. In contrast to Komazawa et al., P-gp was found to be significantly reduced [144].

#### Adult CKD patients

Contrary to the Intact Nephron Hypothesis, it has been shown that, depending on the underlying cause of CKD, active secretion can increase relative to glomerular clearance and does not necessarily show a decline in parallel with the decline in glomerular filtration [118, 145]. For instance, in patients suffering from glomerulonephritis, drug clearance may be maintained relative to the reduced GFR by preservation of active tubular secretion [146]. Hsueh et al. reviewed clinical studies regarding the inhibition of OAT1 and OAT3 in CKD patients, and their data suggest that uremic solutes contribute to the decline in renal drug clearance in CKD patients by inhibition of OAT1 and OAT3 [147]. One may hypothesize that reduction in uptake transporters due to uremic toxins may lead to increased circulating drug levels in human as well.

### Tubular reabsorption

Most of the 120 ml/min of plasma water filtered at the glomerulus is reabsorbed during its passage through the renal tubule and in the end only about 1–2 ml/min appears as urine. Reabsorption of water occurs along the entire nephron, yet, the majority is reabsorbed in the proximal tubule [148]. As plasma water is reabsorbed, a concentration gradient appears between drug in the tubules and unbound drug in the blood. For the majority of drugs and drug metabolites, tubular reabsorption takes place by passive diffusion. If the drug is able to pass through the membranes of the tubular cell, it moves down this concentration gradient and is reabsorbed from the tubular fluid back into the blood. Many vital endogenous compounds, including vitamins, electrolytes, and amino acids, are actively reabsorbed via transporters [148]. Several transporters, such as OAT4, urate transporter 1 (URAT1), peptide transporter 2 (PEPT2), organic cation, and carnitine transporters OCTN1 and OCTN2, reabsorb selected compounds [135]. In line with the maturation of transporters involved in tubular secretion, little data are available regarding the ontogeny of transporters involved in tubular reabsorption, as reviewed by Brouwer et al. [113].

### Renal metabolism

As CYP enzyme activity in the human kidney homogenate is about 14–18% of hepatic enzyme activity [149, 150], renal impairment could affect renal drug metabolism.

#### Impact of age in non CKD children

The administration of ifosfamide for the treatment of solid tumors in children may illustrate the ontogeny of renal metabolism. Ifosfamide is metabolized into the toxic metabolite chloroacetaldehyde [151]. Aleksa et al. investigated the expression of CYP3A expression in pigs, showing low levels in early life, followed by a significant increase in both CYP3A expression and ifosfamide metabolism with age followed by a decrease to adult levels [152]. Subsequently, ontogeny in renal CYP3A enzyme activity may explain why younger children (median age 2.2 years) treated with ifosfamide experience more severe nephrotoxicity compared to older children [153].

#### Adult CKD patients

The active form of vitamin D, calcitriol (1,25-dihydroxycholecalciferol vitamin D3), is taken by patients with CKD to increase calcium absorption and prevent bone disease. Metabolic activation of vitamin D (from diet or synthesized in the skin) to calcitriol requires hydroxylation of 25-hydroxycholecalciferol at the 1alpha-position in the kidney [154]. Therefore, in patients with CKD, it may be better to administer vitamin D in the form of calcitriol or 1a-hydroxycholecalciferol [6].

## Drug dosing

As summarized in this review, several pharmacokinetic parameters may be altered in CKD patients. The bioavailability of certain drugs may be decreased due to increased gastric pH and formation of insoluble salts, necessitating a dose increase. In contrast, for other drugs, bioavailability may be increased due to downregulation of transporters and enzymes in enterocytes, and a lower dose should be administered. Alterations in protein and tissue binding and body composition may impact the volume of distribution, requiring an increase or decrease in dose. Furthermore, CKD may have various effects on drug metabolizing enzymes and transporters. A well-known effect of CKD is a decreased GFR. However, depending on the cause of renal dysfunction, tubular secretion can be affected to a variable extent. Individual patient and drug characteristics should be taken into account when proposing an alternative, individual dosing regimen. In children, developmental changes cause variations in absorption, distribution, metabolism, and excretion over time. All changes should be taken into account when selecting and dosing drugs in children.

Taken together, it is difficult to develop generic drug dosing guidelines for pediatric CKD patients due to a large variability in PK changes in CKD and maturational changes in children. Both sub- and supratherapeutic dosing can occur when the appropriate dose adjustments are not made in (pediatric) patients with kidney disease. Subtherapeutic dosing increases the risk of treatment failure; supratherapeutic dosing increases the risk of toxicity. A practical approach to adjusting drug doses in CKD is to assume that renal drug clearance will decrease in proportion to GFR and that nonrenal clearance is unchanged, which is also known as the Dettli method [155, 156]. However, this would ignore the role of other processes involved in PK, including the alteration of the functional expression of numerous drug metabolizing enzymes and drug transporters and tubular function [4]. In children, PK may be different from adult healthy and CKD patients due to growth and development and the underlying changes in the processes involved in absorption, distribution, metabolism, and excretion. Linear extrapolation of doses from adults will lead to under- or overdosing, dependent on the age of the child and the relevant disposition pathways of the drug. In pediatrics, dose adjustments are undertaken to obtain adequate exposure and pharmacodynamic effects. Still, to date, for half of all drugs, high-quality data are lacking to support the optimal effective and safe drug dose in children, as reflected by the percentage of drugs being prescribed off-label to children [157]. In a project by the Dutch Pediatric Formulary, we found that evidence to support dosing guidelines in pediatric patients, with CKD, remains especially limited (unpublished data). Consequently, current dosing guidance in pediatric patients with CKD in the Dutch Pediatric Formulary and other dosing guidelines, such as the online Pediatric Drug Handbook, is derived from adult data and often excludes young infants and neonates [158, 159].

Nevertheless, although drug-specific data are lacking in this vulnerable population, a few basic principles can be kept in mind to guide dosing adjustments in children with CKD. The important principles to consider include the therapeutic index of the drug, the presence of active metabolites that are eliminated by the kidneys, and the extent of reduction in kidney function. In Table 1, dosing advice for pediatric CKD patients is given for a few drugs, on the basis of the PK processes affected (ADME). This table includes both commonly prescribed drugs and drugs that are illustrative of one of the specific PK processes.

**Table 1:** Dosing advice for pediatric CKD patients for 18 different drugs, on the basis of the PK processes affected

Drug	Vd (L/kg)	PPB (%)	Mode of elimination	Effect CKD	T ½ N (h)	T ½ ESKD (h)	Required dosing GFR < 30 ml/min/1.73 m^2^	Required dosing GFR < 15 ml/min/1.73 m^2^	Ref
Absorption									
Propranolol	4	80–95	Considerable first pass effect in the liver, almost completely excreted in the urine as active and inactive metabolites, < 5% unchanged	Increased bioavailability, accumulation active metabolites	2–6	Unchanged	Start with small dose, titrate to response*	Start with small dose, titrate to response*	[167, 168]
Amitriptyline	6–36	96	Extensive first pass metabolism, almost completely excreted in urine, 5% unchanged.	Increased bioavailability, accumulation active metabolites	9–25	Unchanged	Reduce dose or increase dosing interval*	Reduce dose or increase dosing interval*	[167, 168]
Ciprofloxacin	2.5	20–40	10–20% metabolized, mostly excreted in urine, 40–70% unchanged	Chelate formation 50% of oral dose is bound when given together with currently used phosphate binders. Decreased renal clearance. Variable increase in elimination of the drug via the transluminal route across the bowel mucosa.	3–5	8	Alter dosing regimen of separate drugs 50–100% of normal dose/increase dose interval	Alter dosing regimen of separate drugs 50% of normal dose/increase dose interval	[158, 159, 167, 168]
Furosemide	0.07–0.2	91–99	20% converted to metabolites. Mostly excreted in the urine, largely unchanged. Excreted by tubular secretion.	Reduced bioavailability due to increased gastric pH. Decreased protein binding, increased Vd. Impaired tubular secretion.	0.5–2	9.7	Normal dose, increased doses may be required*	Normal dose, increased doses may be required*	[159, 167, 168]
Distribution									
Oxazepam	0.6–1.6	85–97	Metabolism by conjugation. Mostly excreted in the urine as inactive metabolites, < 1% excreted unchanged.	Decreased protein binding, increased Vd	3–21	25–90	Normal dose	Start at low dose, increase according to response*	[167, 168]
Phenytoin	0.52–1.19	90	Extensive biotransformation in the liver, mostly excreted in the bile.	Decreased protein binding	7–42	Unchanged	Normal dose, request free phenytoin concentrations after 4–5 days	Normal dose, request free phenytoin concentrations after 4–5 days	[158, 159, 167, 168]
Pravastatin	0.5	50	Biotransformation in the liver, mostly excreted in the feces.	Increase in Vd, due to fluid retention	1.5–2	Unchanged	Starting dose 10 mg	Starting dose 10 mg	[158, 167]
Metabolism									
Captopril	2	25–30	40% hepatic metabolism, 65% excretion via urine, 40–50% unchanged.	Decreased clearance	2.3	21	Start low, titrate to response. 75% of normal dose	Start low, titrate to response. 50% of normal dose	[159, 167, 168]
Repaglinide	0.4	>98	Hepatic CYP3A4 metabolism to inactive metabolites, excretion via bile.	Decreased clearance	1	2	Start low, titrate to response*	Start low, titrate to response*	[167, 168]
Reboxetine	26-63 L	97	Hepatic CYP3A4 metabolism to inactive metabolites, elimination in urine, 10% unchanged	Decreased clearance	13	26	50% of initial dose, adjust according to response	50% of initial dose, adjust according to response	[167, 168]
Renal metabolism									
Insulin	0.15	5	Hepatic and renal metabolism, 1–1.5% unchanged excretion in urine	Decreased clearance	2–5	13	Variable, based on glucose levels.	Variable, based on glucose levels.	[168]
Vitamin D	–	–	Renal metabolism	No conversion to active form	–	–	Use active form	Use active form	[167]
Excretion									
Benzyl-penicillin	0.5–0.6	60	Almost completely excreted unchanged in urine.	Decreased clearance	0.5	10	50%, dose interval 12 h	25–50%, dose interval 12-16 h	[167, 168]
Aciclovir	0.7	9–33	Predominantly excreted in urine, > 80% unchanged	Decreased clearance	2.9	19.5	100% dose, dose interval 24 h	50% dose, dose interval 24 h	[158, 159, 167, 168]
Fluconazole	0.65–0.7	11–12	> 90% excreted in the urine, 80% unchanged.	Decreased clearance	30	98	50% of normal dose, dose interval 24 h	50% of normal dose, dose interval 24-48 h	[158, 159, 167, 168]
Morphine	3–5	20–35	Hepatic conjugation, 10% excreted unchanged in urine	Decreased clearance. Accumulation of active metabolites (morphine-6-glucuronide, morphine-3-glucuronide)	2.5 Active metabolite 3–5	Unchanged Active metabolite 50	Small doses, extended dosing intervals, titrate to response. 75% of normal dose. Consider switch to alternative drug (e.g., piritramide)	Small doses, extended dosing intervals, titrate to response. 50% of normal dose. Consider switch to alternative drug (e.g., piritramide)	[158, 159, 167, 168]
Vancomycin	0.47–1.1	10–50	80–90% excreted unchanged in urine	Decreased clearance	6	120–216	100% of normal dose, increase dose interval to 48–72 h	100% of normal dose, increase dose interval to 1 week	[158, 159, 167, 168]
Amikacin	0.22–0.29	< 20	94–98% excreted unchanged in urine	Decreased clearance	2–3	17–150	Dose reduction and increase dose interval based on drug levels*	Dose reduction and increase dose interval based on drug levels*	[158, 159,167, 168]
Gentamicin	0.3	0–30	90% excreted unchanged in urine	Decreased clearance	2–3	20	Dose reduction based on drug levels, increase dose interval to 48 h*	Dose reduction and increase dose interval based on drug levels*	[158, 159,167, 168]
Tobramycin	0.25	< 5	90% excreted unchanged in urine	Decreased clearance	2–3	5–70	Dose reduction based on drug levels*	Dose reduction and increase dose interval based on drug levels*	[158, 159,167, 168]

*CKD* chronic kidney disease, *ESKD* end-stage kidney disease, *GFR* glomerular filtration rate, *h* hours, *N* normal, *ND* no data, *PPB* plasma protein binding, *Ref* references, *T½* half-life, *Vd* volume of distribution

*No exact dose recommendations or contradictory dose recommendations are available in the literature

### Basic principles of dose adjustments in CKD

**Fig. 4:**
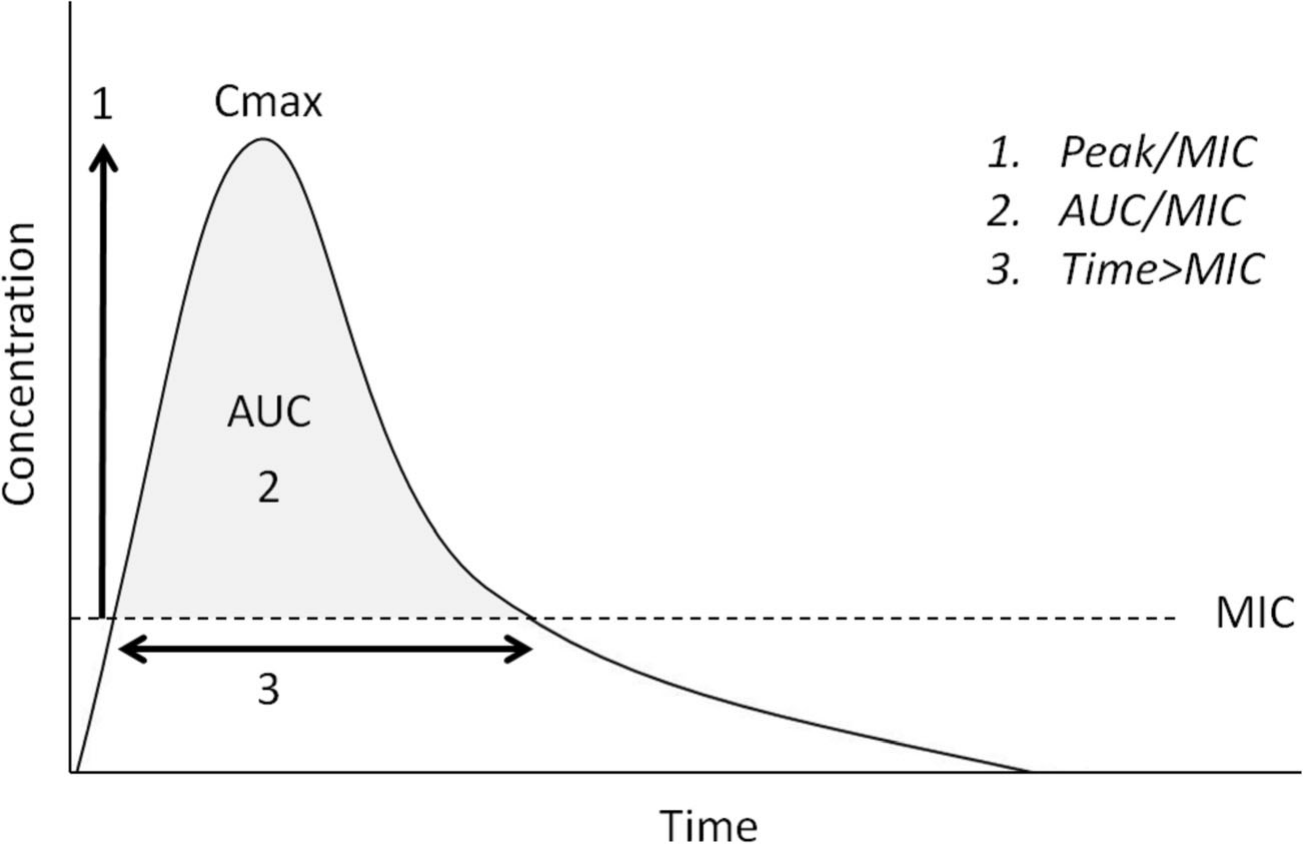
Concentration-time profile of antibiotics. Peak/MIC: The ratio of maximum free drug plasma concentration to the MIC. AUC/MIC: The ratio of the total exposure of the drug to the MIC. Time/MIC: The proportion of time that the plasma concentration exceeds the MIC. AUC area under the concentration time curve, Cmax maximum concentration, MIC minimum inhibitory concentration for a pathogen, T time

Drug exposure relates to the maximum plasma concentration and/or the area under the concentration time curve (AUC). In general, supra-therapeutic exposures increase the risk of dose-related adverse drug reactions, and subtherapeutic exposure increases the risk of ineffective therapy. As infections are common in patients with CKD, basic knowledge on the pharmacodynamic properties of antibiotics is necessary to optimize treatment, as was recently reviewed by Momper et al. [160]. For antibiotics, three PK-PD targets describe features of the concentration-time profile that maximize antibiotic efficacy (Fig. 4):

1. The ratio of maximum free drug plasma concentration to minimum inhibitory concentration (MIC) (aminoglycosides)
2. The ratio of AUC to MIC (vancomycin)
3. The proportion of time that the plasma concentration exceeds the MIC (β lactam antibiotics)

For each individual drug, the PK-PD target should be taken into account when prescribing the dosing regimen. Dose adjustments for primarily hepatically metabolized drugs should be carefully considered as well, as their pharmacologically active and/or toxic metabolites can be excreted by the kidney, e.g., morphine and mycophenolate mofetil [161, 162]. The minimum change in kidney function that requires a change in dose is not well defined [5]. In general, dose adjustment is unlikely to be required when < 30% of the dose is excreted by the kidney [5, 163]. However, in drugs with a small therapeutic index, this statement should be reconsidered.

### Loading dose

Some drugs and clinical situations, for instance antibiotics in patients with a severe infection, require rapid therapeutic concentrations. The time to reach steady state is determined by the half-life (*T*_1/2_), and it takes three to five half-life periods to reach steady state. Half-life is a PK parameter determined by both clearance (CL) and Vd (*T*_1/2_ = 0.693 × Vd/CL). When *T*_1/2_ is prolonged, the time to reach steady state increases proportionally. Hence, for some drugs, a loading dose is necessary to decrease the time needed to reach the plateau drug concentration. The loading dose to achieve a target concentration is determined by the Vd (loading dose = target concentration × Vd). However, when CKD coincides with an altered volume of distribution of a drug, the loading dose must be modified [134].

### Maintenance dose

The maintenance dose is aimed at maintaining the desired steady-state drug concentrations. The maintenance dose is determined by the drug concentration at steady state (Css) and the CL of the drug from the body (maintenance dose = Css × CL). For intermittent dosing, the desired dosing interval should be taken into account as well. A decrease in drug clearance with kidney disease necessitates, therefore, a decrease in either maintenance dose, an increase in the dosing interval, or both [34]. The dosing frequency depends on the toxicity profile of the drug. An important question to consider is whether the effects of the drug relate to the peak or to average exposure of the drug. A relatively long dosing interval will require a relatively high Cmax to maintain an acceptable mean drug concentration or AUC. Therefore, in most instances, a reduction in dose rather than an increase in dosing interval is appropriate, with the exception of drugs where high peak serum concentrations are beneficial such as gentamicin and tobramycin.

## Research priorities

As stated in the KDIGO guideline on CKD evaluation and management, national and international research groups should ensure adequate representation of adult CKD patients in clinical trials to improve the understanding of PK and PD parameters in this population [164]. Moreover, drug research involving pediatric indications and drug dosing optimization in pediatric clinical practice is challenging. Limited observational data can be used to create dosing advice for specific drugs and patient categories, as we previously demonstrated [165]. Furthermore, physiologically based pharmacokinetic (PBPK) modeling can be used to predict the pharmacokinetic behavior of drugs in humans using preclinical data [166]. For the pediatric (CKD) population, we believe that PK studies and PBPK modeling should be used to incorporate pediatric developmental physiology and disease to predict drug exposure in vulnerable patient groups. Currently, several initiatives are in progress to determine the population PK of, for instance, antibiotics (NCT03248349, NCT02539407) and antiretroviral drugs (NCT03194165) in the pediatric (non-CKD) population.

## Conclusion

CKD is a heterogeneous condition, which is a major public health problem worldwide. In patients with CKD, the PK of several drugs can be significantly altered. For pediatric CKD patients, the impact of maturation on drug disposition and action should be taken into account as well. As shown in this review, the effects may be variable and are not limited to renal drug clearance. In fact, CKD may also have a major influence on drug absorption, distribution, and drug metabolism in the liver, gut, and kidneys. Inappropriate dose adjustments may lead to sub- or supratherapeutic concentrations predisposing the patient to either therapeutic failure or adverse drug reactions. As general guidelines in pediatric CKD patients are lacking, prescribing an appropriate dose requires knowledge on specific PK alterations in this study population. Finally, we believe that all available data should be used to extrapolate dosing advice in the adult population to the pediatric CKD population.

## Multiple choice questions

1. Which of the following pharmacokinetic processes may be altered in patients with CKD?

- Absorption
- Distribution
- Metabolism
- Elimination
- All of the above

2. In case of CKD, the dose of a drug should be reduced.

- Yes
- No
- Depends on the drug given

3. Where does metabolism take place in the body?

- Liver
- Kidneys
- Gut
- All of the above

4. Which dose should be adjusted in case of a decreased GFR?

- Loading dose
- Maintenance dose
- Both

5. All drug metabolizing enzymes will increase with age

- True
- False
